# The Diagnosis and Prognosis Value of Circulating Exosomal lncRNA MALAT1 and LNC_000226 in Patients With Acute Myocardial Infarction: An Observational Study

**DOI:** 10.1002/iid3.70088

**Published:** 2024-12-06

**Authors:** Xiaodong Gu, Jingyuan Hou, Ruiqiang Weng, Jiawei Rao, Sudong Liu

**Affiliations:** ^1^ Meizhou Clinical Institute Shantou University Medical College Meizhou China; ^2^ Research Experimental Center Meizhou People's Hospital (Huangtang Hospital) Meizhou China; ^3^ Guangdong Engineering Technology Research Center of Molecular Diagnostics for Cardiovascular Diseases Meizhou China; ^4^ Meizhou Clinical Medical School Guangdong Medical University Meizhou China

**Keywords:** acute myocardial infarction (AMI), exosome, lncRNA

## Abstract

**Background:**

Acute myocardial infarction (AMI) stands as a leading cause of global morbidity and mortality. This study aims to explore the potential roles of circulating exosomal lncRNA MALAT1 and LNC_000226 in AMI diagnosis and prognosis.

**Methods:**

This retrospective observational study included 90 patients with AMI and 88 patients with normal coronary artery (NCA). Plasma exosomes were isolated via ultracentrifugation, and the levels of exosomal lncRNA MALAT1 and LNC_000226 were examined using qRT‐PCR. Major adverse cardiovascular events (MACEs) that occurred during 1‐year follow‐up post‐stent implantation were collected. The diagnostic value of exosomal MALAT1 and LNC_000226 was determined by receiver operating characteristic (ROC) analysis. The association between exosomal LNC_000226 and MACEs was assessed by Kaplan–Meier and Cox regression analysis.

**Results:**

Both lncRNA MALAT1 and LNC_000226 levels in plasma exosomes were elevated in AMI patients compared to NCA controls. Moreover, LNC_000226 (AUC: 0.889, sensitivity: 82%, specificity: 72%) exhibited superior diagnostic performance compared to MALAT1 (AUC: 0.707, sensitivity: 71%, specificity: 57%). During 1‐year follow‐up period, the incidence of MACEs was significantly higher among patients with high exosomal LNC_000226 levels compared to those with low exosomal LNC_000226 levels [64% (29/45) vs. 40% (18/45), *p* < 0.05]. Multivariable Cox regression analysis revealed a positive association between exosomal LNC_000226 level and the risk of MACEs in AMI patients (HR: 1.959, 95% CI: 1.040–3.689).

**Conclusion:**

Circulating exosomal lncRNA MALAT1 and LNC_000226 are promising biomarkers for diagnosing AMI, with LNC_000226 potentially indicating a prognosis.

## Graphical abstract

Acute myocardial infarction (AMI) stands as a leading cause of global morbidity and mortality. Emerging evidence has shown that circulating exosomes are promising diagnostic tools for AMI. The present study revealed circulating exosomal lncRNA MALAT1 and LNC_000226 are promising biomarkers for diagnosing AMI, with LNC_000226 potentially indicating a prognosis.

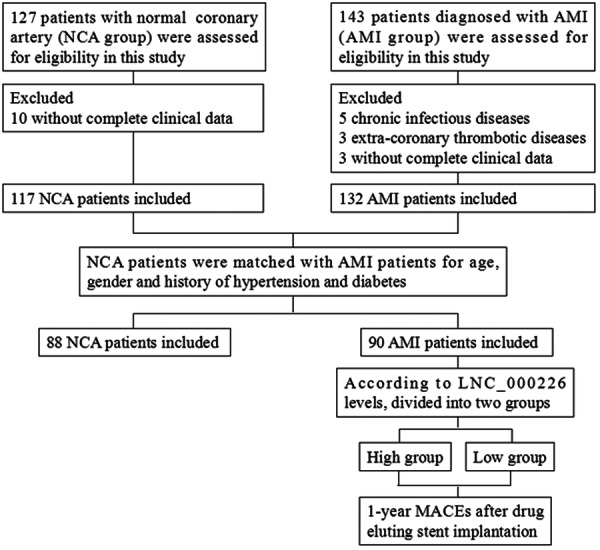

**Abbreviations**
AMI: acute myocardial infarction
CVD: cardiovascular disease
lncRNAs: long noncoding RNAs
MACEs: major adverse cardiovascular events

## 1 Introduction

Acute myocardial infarction (AMI), as an acute and severe form of ischemic heart disease, has drawn significant attention due to its high morbidity, mortality, and adverse prognosis [1]. In China, AMI increasingly poses a significant health threat and burden on healthcare as entering an aging society. It was projected that the incidence of AMI cases in China would rise from 8 million in 2010 to 23 million by 2030. Current interventions, e.g. anti‐atherosclerotic, antithrombotic therapies, have been shown to reduce the incidence of AMI by 30% to 45% [2]. The implementation of percutaneous coronary intervention (PCI) has significantly reduced mortality rates associated with AMI. However, the occurrence of major adverse cardiovascular events (MACEs) following treatment represents a significant threat to the health and quality of life of patients with AMI [3]. Therefore, identifying effective biomarkers that can improve AMI diagnosis and prognosis is urgent for AMI management.

Although cardiac troponins (cTnI) are currently the most widely used plasma biomarkers for AMI, their diagnostic efficacy may be influenced by various factors like age, genetics other diseases. Therefore, there is a pressing necessity for the development of novel, noninvasive, and highly sensitive biomarkers to precisely characterize the patient's disease state. Long noncoding RNAs (lncRNAs) are a group of noncoding RNAs of > 200 nucleotides in length with a lack of protein‐coding potential [4]. Due to their pronounced tissue specificity and the presence of complex and stable secondary structures, lncRNAs have been recognized by researchers as a promising class of biomarkers [5]. Accumulating evidence suggests that lncRNAs play a regulatory role in gene expression at multiple levels and are closely associated with the pathophysiology of cardiovascular diseases [6]. Some studies have demonstrated that lncRNA ANRIL regulate the proliferation and apoptosis of vascular smooth muscle cells (VSMCs) and macrophages, thus participant in the development of atherosclerotic plaques; increased expression of ANRIL transcripts was correlated with atherosclerosis risk [7, 8, 9]. LncRNA MHRT was a heart‐specific, cardioprotective antisense lncRNAs localized within myosin heavy chain 7 (Myh7) locus, and reduced expression of lncRNA MHRT in cardiac tissue has been associated with the accelerated progression of cardiac hypertrophy and heart failure [10]. Therefore, altered lncRNAs in circulation would be potential diagnostic, therapeutic, and prognostic biomarkers for AMI.

Exosomes are small extracellular vesicles (< 150 nm in diameter) that are actively released by cells under both physiological and pathological conditions. Exosomes function as intercellular transport messengers by carrying a variety of biological molecules, including proteins, RNA, and DNA [11]. Researchers become increasingly interested in the role of exosomes in cardiovascular diseases, particularly as signaling mediators, biomarkers, and potential therapeutic targets [12, 13]. Given that exosomes are the primary source of plasma lncRNAs, the function of exosomal lncRNAs is frequently subjected to analyzed [14]. Kenneweg F et al. discovered that exosomal lncRNAs derived from hypoxic cardiomyocytes enter fibroblasts, thereby inducing the expression of profibrotic genes [15]. Huang et al. reported that exosomes harvested from mesenchymal stem cells pretreated with atorvastatin demonstrate cardioprotective properties, mediated through the activity of lncRNA H19 [16]. Another study has demonstrated the levels of circulating exosomal lncRNA‐UCA1, lncRNA‐NEAT1, and lncRNA‐MMP9 were increased in AMI patients [17], indicating the potential of exosomal lncRNAs in predicting and diagnosing AMI [18]. In our previous work, we found that the expression of lncRNA MALAT1 and LNC_000226 was significantly increased in peripheral blood mononuclear cells (PBMCs) of unstable angina patients comparing to those of normal coronary artery (NCA) controls [19]. However, the expression of MALAT1 and LNC_000226 in exosomes remains unknown.

This research project intends to investigate the diagnostic and prognostic significance of exosomal MALAT1 and LNC_000226 in AMI. This study will deepen our understanding of the biological functions of exosomes in cardiovascular diseases.

## 2 Materials and Methods

### 2.1 Study Population

**Figure 1:**
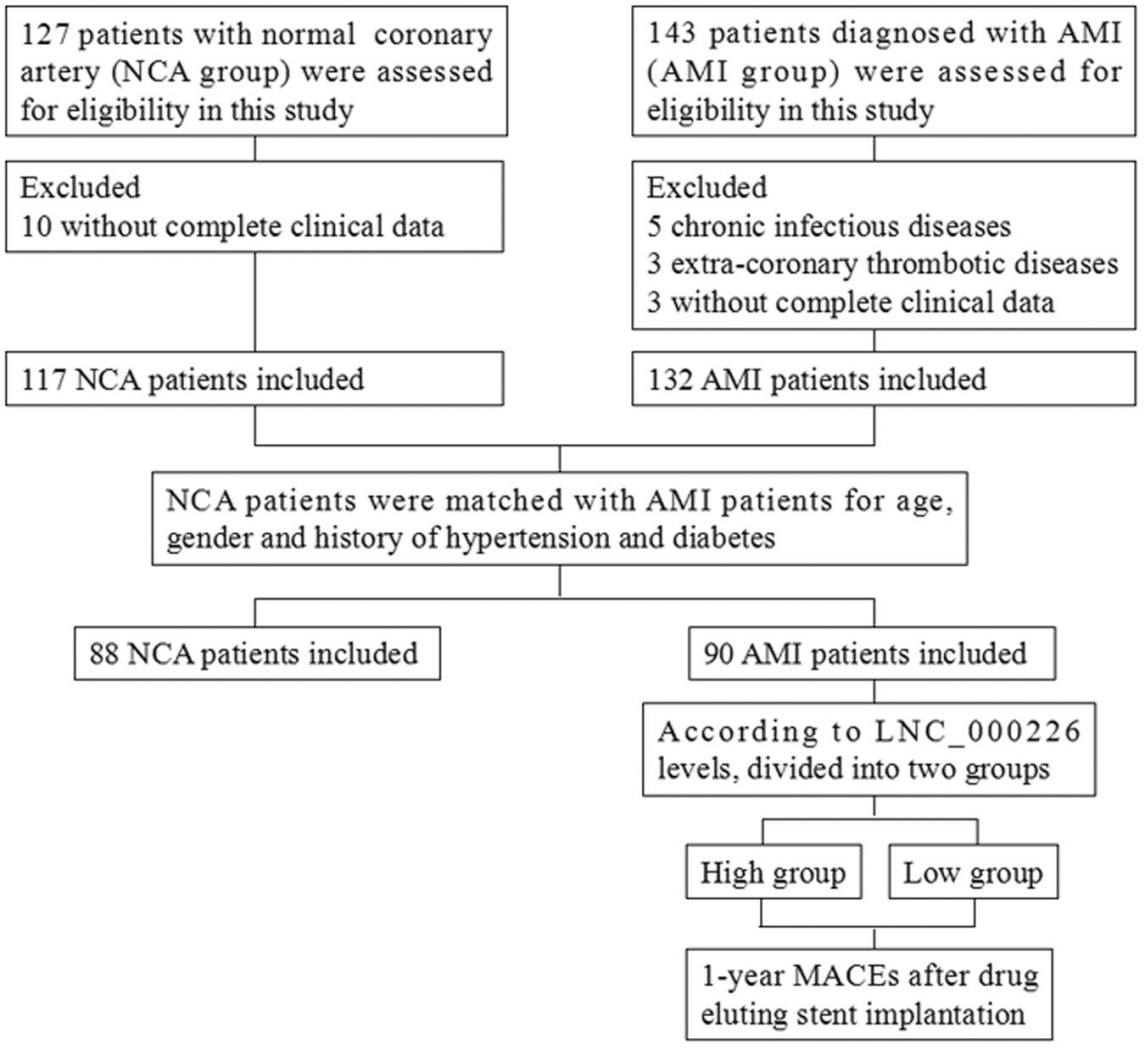
Flow chart of patient enrollment.

This retrospective observational study was approved by the Ethics Committee of Meizhou People's Hospital (No. MPH‐HEC 2021‐CY‐17). The study enrolled patients diagnosed with AMI from the Center for Cardiovascular Disease of Meizhou People's Hospital, adhering to the diagnostic criteria for AMI established by the American Heart Association (AHA) and the American College of Cardiology (ACC). All AMI patients underwent successful drug‐eluting stent implantation. Control patients with normal coronary arteries (NCA group) were selected from the Center for Cardiovascular Disease. Coronary angiography confirmed the status of coronary arteries. Patients with cardiomyopathy, myocarditis, acute and chronic infectious diseases, extracoronary thrombotic diseases, and incomplete data were excluded. AMI patients and NCA patients were matched for sex, age, and history of hypertension and diabetes. All participants provided written consent before their involvement in the study. A flowchart depicting patient enrollment is presented in Figure 1.

### 2.2 Clinical Data Collection and Follow‐Up

The characteristics of AMI and NCA patients were collected from an electronic medical record system (DHC Mediway Technology Co., Ltd, Beijing, China). The high‐expression LNC_000226 group comprised AMI patients with levels exceeding the median LNC_000226 level in the AMI group. The remaining AMI patients were categorized into the low‐expression LNC_000226 group. Major cardiovascular events (MACEs) within 1‐year post‐stent implantation included refractory angina, recurrent AMI, unplanned PCI, stent thrombosis, cardiogenic cerebral infarction, and cardiac death. One‐year follow‐up data on MACEs in AMI patients were obtained from the electronic medical record system and via telephone interviews.

### 2.3 Isolation and Identification of Plasma Exosomes

During emergency admissions, blood samples were obtained from individuals and collected in EDTA anticoagulated tubes. Following centrifugation at 2000 g for 15 min, plasma was separated, and the supernatants were transferred to new tubes for the isolation of plasma exosomes. Plasma exosomes were subsequently isolated utilizing ultracentrifugation. The supernatant was combined with an equal volume of PBS, followed by centrifugation at 2000 g for 10 min at 4°C. The resultant supernatant was carefully transferred to ultracentrifuge tubes and spun at 200,000 g for 30 min at 4°C. Subsequently, the supernatant was decanted, and the exosomes were resuspended in 100 uL of PBS. Electron microscopy (JEM‐1400, JEOL, Japan) was used to observe the exosomes, while nanoparticle tracking analysis (Zetaview, Particle Metrix, Germany) was employed to measure their particle diameter. Protein markers in the exosomes, such as CD63 and TSG101, were assessed utilizing a western blot.

### 2.4 Reverse Transcription‐Quantitative Polymerase Chain Reaction (qRT‐PCR)

Exosomal RNA was isolated utilizing TRIzol reagents (Invitrogen, Carlsbad, CA, USA), followed by mRNA expression analysis through qRT‐PCR utilizing the Prime Script RT reagent kit and TB GreenTM Premix Ex TaqTM II kit (Takara Bio, Tokyo, Japan). Relative mRNA expression was normalized to GAPDH. The primer sequences for qRT‐PCR were as follows: lncRNA MALAT1 forward, 5′‐ ATGAGCCACTGGGTGTACCA ‐3′; lncRNA MALAT1 reverse, 5′‐ CAGACCAACCCCCAGTTCAA ‐3′; LNC_000226 forward, 5′‐ CTTTACGACGATAGGCCTC ‐3′; LNC_000226 reverse, 5′‐ CACACTTTCTGTAGAATCTG ‐3′; GAPDH forward, 5′‐ ATGACATCAAGAAGGTGGTG ‐3′; GAPDH reverse, 5′‐ CATACCAGGAAATGAGCTTG ‐3′.

### 2.5 Statistical Analysis

Data were reported as mean ± SD, number (percentage), or median (interquartile range). The Shapiro–Wilk test was utilized to detect whether the data follows a normal distribution. Group comparisons were made utilizing Student's *t*‐test, Mann–Whitney U test, and Chi‐squared (χ^2^) test as appropriate. Receiver operating characteristic (ROC) curve analysis was utilized to evaluate the diagnostic performance of lncRNA. The link between potential predictors and 1‐year MACEs in AMI patients was determined by multivariable Cox regression analysis (enter model). One‐year MACE‐free survival curve was analyzed utilizing Kaplan–Meier analysis and log‐rank test. Statistical analyses were carried out utilizing SPSS 20.0 software (IBM Corp., Armonk, NY, USA), with significance set at *p* < 0.05.

## 3 Results

### 3.1 Identification of Plasma Exosomes

**Figure 2:**
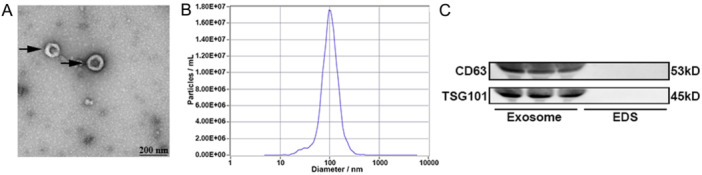
Isolation and identification of plasma exosomes (A) The observation of plasma exosomes using electron microscopy, Scale bar: 200 nm. (B) The particle diameter of plasma exosomes was measured using nanoparticle tracking analysis. (C) The protein levels of exosome markers in plasma exosomes were detected by western blot. EDS, Exosome‐depleted supernatants.

Plasma exosomes were characterized utilizing transmission electron microscopy, nanoparticle tracking analysis, and western blot analysis techniques. Transmission electron microscopy revealed the presence of exosomes exhibiting a circular double‐layer membrane bubble structure (Figure 2A). Nanoparticle tracking analysis indicated that the diameter of exosomes was found to be around 100 nm (Figure 2B). Western blot analysis confirmed the expression of specific proteins CD63 and TSG101 in exosomes, while these proteins were not detected in exosome‐depleted supernatants (EDS) (Figure 2C).

### 3.2 The Diagnosis Value of Exosomal lncRNa MALAT1 and LNC_000226 in AMI

**Figure 3:**
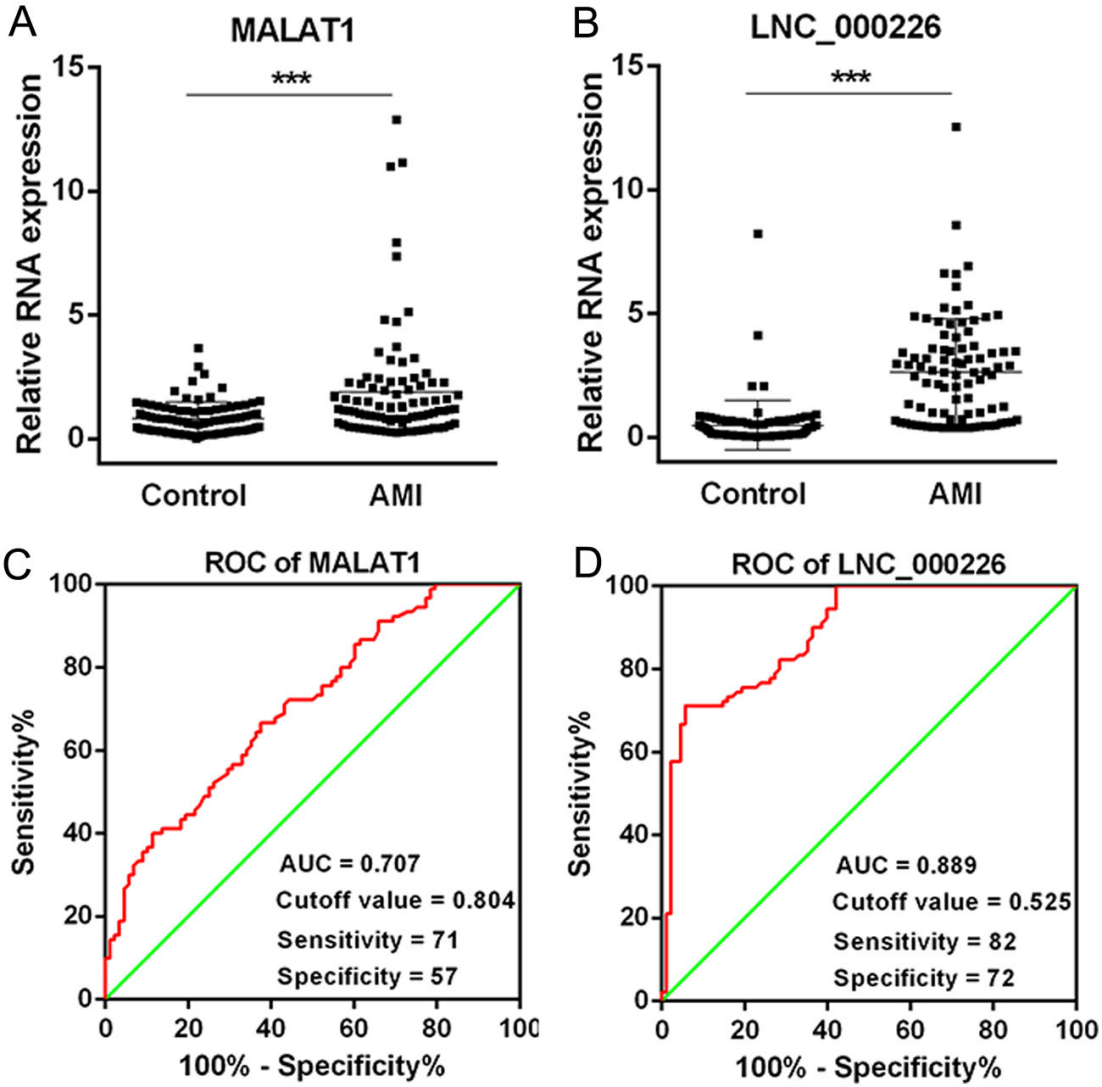
The diagnosis values of exosomal lncRNA MALAT1 and LNC_000226 in AMI patients The expression of exosomal lncRNA MALAT1 (A) and LNC_000226 (B) in AMI patients and the control patients. The diagnostic value of exosomal lncRNA MALAT1 (C) and LNC_000226 (D) in AMI and control patients.

The baseline demographic and clinical characteristics of AMI patients (*n* = 90) and NCA controls (*n* = 88) are outlined in Table 1. There were no significant differences between AMI and NCA groups regarding age, gender, history of hypertension/diabetes/dyslipidemia, smoking status, and drinking status (all *p* > 0.05). The expression of lncRNA MALAT1 and LNC_000226 in exosomes was detected using qRT‐PCR assays. Our data showed that both exosomal MALAT1 and exosomal LNC_000226 were significantly higher in AMI patients compared with NCA patients (all *p* < 0.05, Figure 3A,B). The AMI diagnostic value of exosomal MALAT1 and exosomal LNC_000226 was evaluated by the receive operator curve (ROC). The results showed that exosomal LNC_000226 demonstrated a better diagnostic value (AUC: 0.889, Sensitivity: 82%, Specificity: 72%) compared to exosomal MALAT1 (AUC: 0.707, Sensitivity: 71%, Specificity: 57%).

**Table 1:** Clinical characteristics of NCA individuals and AMI individuals.

Variables	Total (*n* = 178)	NCA (*n* = 88)	AMI (*n* = 90)	*P*‐values
Age (years)	61.7 ± 9.7	60.7 ± 8.9	62.6 ± 10.5	0.187
Male, n (%)	132 (74)	61 (69)	71 (79)	0.145
Hypertension, n (%)	71 (40)	33 (38)	38 (42)	0.520
Diabetes, n (%)	29 (16)	11 (13)	18 (20)	0.17
Dyslipidemia, n (%)	52 (29)	27 (31)	25 (28)	0.670
Smokers, n (%)	57 (32)	28 (32)	29 (32)	0.954
Drinker, n (%)	7 (3.9)	3 (3.4)	4 (4.4)	0.722
TG (mmol/L)	1.90 ± 1.42	1.67 ± 1.27	2.12 ± 1.52	0.036
TC (mmol/L)	4.68 ± 1.15	4.75 ± 0.99	4.62 ± 1.29	0.459
HDL‐C (mmol/L)	1.27 ± 0.82	1.35 ± 0.32	1.20 ± 1.10	0.213
LDL‐C (mmol/L)	2.63 ± 0.89	2.55 ± 0.74	2.71 ± 1.02	0.217
cTnI (ng/L)	0.004 (0.057)	0.001 (0.003)	0.031 (2.108)	< 0.001
Lipid‐lowering drug, *n* (%)	125 (70)	35 (40)	90 (100)	< 0.001
Antihypertensive drug, *n* (%)	129 (73)	47 (53)	82 (91)	< 0.001
Antiplatelet drug, *n* (%)	123 (69)	35 (40)	88 (98)	< 0.001

*Note:* Data are expressed as the mean ± standard deviation (SD), number (percentage) or median (interquartile range).

Abbreviations: HDL‐C: high‐density lipoprotein cholesterol; LDL‐C, low‐density lipoprotein cholesterol; LVEF, left ventricular ejection fraction; TC, total cholestero; TG, Triglycerides.

### 3.3 The Prognostic Value of Exosomal LNC_000226 in AMI Patients

**Figure 4:**
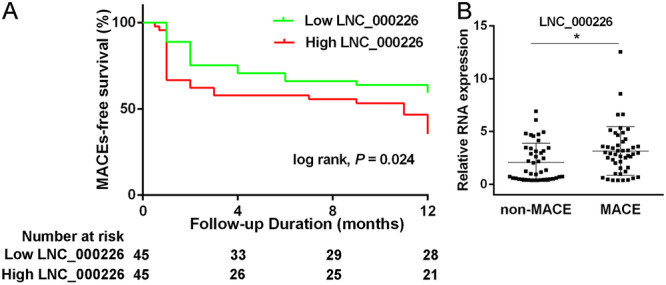
The prognostic value of exosomal LNC_000226 in AMI patients (A) Kaplan–Meier curves for 1‐year MACEs‐free survival in high LNC_000226 level group and low LNC_000226 level group among AMI patients. (B) The expression of exosomal LNC_000226 in non‐MACE group and MACE group.

We further explore whether the expression of exosomal LNC_000226 could predict MACEs in AMI patients after PCI treatment. AMI patients were categorized into high LNC_000226 group (*n* = 45) and low LNC_000226 group (*n* = 45) based on exosomal LNC_000226 expression. During a median follow‐up of 1 year, there were a total of 43 MACEs, with 18 events (40%) among patients with low LNC_000226, and 29 (64%) in patients with high LNC_000226. The incidence of MACEs was significantly higher in AMI patients with high exosomal LNC_000226 levels than those with low exosomal LNC_000226 levels (*p* < 0.05). Although the incidence of refractory angina, recurrent MI, stent thrombosis and cardiogenic cerebral infarction was higher in AMI patients with high exosomal LNC_000226 level compared to those with low exosomal LNC_000226 level, the difference did not reach statistical significance. Kaplan‐Meier analysis and log‐rank test were utilized to assess the 1‐year MACEs‐free survival rate between high LNC_000226 and low LNC_000226 groups (Table 2). The results indicated that higher exosomal LNC_000226 expression was linked to a lower incidence of MACEs‐free survival (*p* = 0.024 Figure 4A). Furthermore, LNC_000226 expression was significantly higher in the MACE group than the non‐MACE group (*p* < 0.05 Figure 4B).

**Table 2:** Clinical characteristics of low LNC_000226 group and high LNC_000226 group in AMI individuals.

Variables	Total (*n* = 90)	Low (*n* = 45)	High (*n* = 45)	*P*‐values
Age (years)	62.6 ± 10.5	60.8 ± 9.7	64.4 ± 11.0	0.099
Male, *n* (%)	71 (79)	38 (84)	33 (73)	0.197
Hypertension, *n* (%)	38 (42)	17 (38)	21 (47)	0.39
Diabetes, *n* (%)	18 (20)	5 (11)	13 (29)	0.035
Dyslipidemia, *n* (%)	245 (28)	13 (29)	12 (27)	0.814
Smokers, *n* (%)	29 (32)	14 (31)	15 (33)	0.822
Drinker, *n* (%)	4 (4.4)	3 (6.7)	1 (2.2)	0.306
cTnI (ng/L)	0.031 (2.108)	0.009 (0.065)	2.090 (12.09)	< 0.001
Disease vessels ≥ 3, *n* (%)	57 (63)	26 (58)	31 (69)	0.274
Stent number (*n*)	1.3 ± 0.6	1.3 ± 0.5	1.5 ± 0.7	0.172
Rapamycin stent, *n* (%)	83 (92)	44 (98)	39 (87)	0.110
MACE, *n* (%)	47 (52)	18 (40)	29 (64)	0.020
Refractory Angina (*n*)	30 (33)	13 (29)	17 (38)	0.371
Recurrent MI (*n*)	1 (1.1)	0 (0)	1 (2.2)	1.000
Unplanned PCI (*n*)	8 (8.9)	4 (8.9)	4 (8.9)	1.000
Stent thrombosis (*n*)	7 (7.8)	1 (2.2)	6 (13)	0.110
Cardiogenic cerebral infarction (*n*)	1 (1.1)	0 (0)	1 (2.2)	1.000
Death (*n*)	0 (0)	0 (0)	0 (0)	—

*Note:* Data are expressed as the mean ± standard deviation (SD), number (percentage) or median (interquartile range).

Abbreviations: MACE, major cardiovascular adverse events; MI, myocardial infarction; PCI, percutaneous coronary intervention.

**Figure 5:**
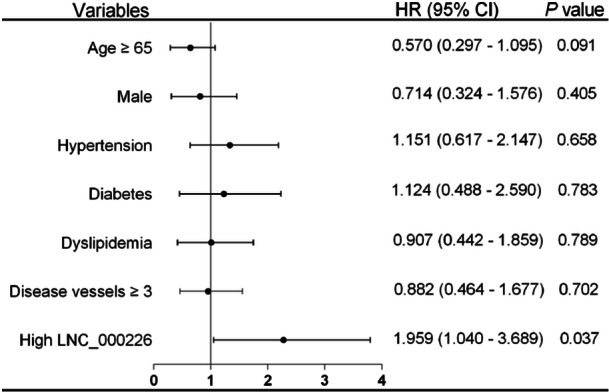
A multivariable Cox regression model for predicting the 1‐year MACEs in AMI patients with stent implantation.

Additionally, a multivariable Cox regression analysis was conducted to assess the prognostic significance of exosomal LNC_000226 in AMI patients. After adjustment for age, sex, hypertension/diabetes/dyslipidemia, and diseased vessels, a strong association was found between high exosomal LNC_000226 levels and the risk of 1‐year MACEs, with an HR of 1.959 (95%CI: 1.040‐3.689, *p* = 0.037; Figure 5).

## 4 Discussion

Exosomes have attracted considerable interest in the cardiovascular field due to their diverse functions in cardiovascular diseases. Existing studies have indicated that exosomal lncRNAs could offer new insights for early AMI diagnosis. In the present study, we examined the expression of lncRNA MALAT1 and LNC_000226 in the exosomes of AMI patients and NCA controls. Our data suggested that circulating exosomal lncRNA MALAT1 and LNC_000226 could potentially function as diagnostic biomarkers for AMI. In addition, exosomal LNC_000226 expression was closely associated with the prognostic outlook of AMI.

Consistent with previous studies [13, 17], the mean age of AMI patients in this study was approximately 60 years, with males accounting for over 70% of the cases. AMI patients and NCA patients included in this study were matched by age, gender, and history of hypertension and diabetes, resulting in largely balanced clinical characteristics between the two groups. PCI is the primary treatment for AMI patients [20], and all AMI patients in this study underwent successful drug‐eluting stent implantation. Therefore, the patients in this study can be considered representative of the population.

Although initially thought to be noise produced during gene transcription, ncRNAs have been reported to play important roles in a variety of diseases and pathophysiological processes. In cardiovascular diseases, some lncRNAs have been established as diagnostic or prognostic biomarkers. Vausort et al assessed five lncRNAs levels in PBMCs of 414 AMI patients and 86 healthy volunteers, and found that lncRNA ANRIL, KCNQ1OT1 and MALAT1 were predictors of left ventricular dysfunction [21]. Kumarswamy R et al. discovered that the plasma level of lncRNA LIPCAR was decreased in the early stages after cardiac event and increased during later phages, and suggesting a strong prognostic value of LIPCAR for detrimental cardiac remodeling and death [22]. Since most of the plasma lncRNAs are presented in extracellular vesicles, researchers turn to study exosomal lncRNAs as potential biomarkers.

Evidence that supports exosomal lncRNAs as invaluable biomarkers of cardiovascular diseases are emerging. Liang et al. reported that circulating exosomal lncRNA SOCS2‐AS1 may be a diagnostic biomarker for coronary artery disease with area under the curve values (AUC) of 0.704 [23]. Hosen MR et al. reported that the expression of circulating exosomal lncRNAs was significantly increased in coronary artery disease patients, and played a pro‐angiogenesis in endothelial cell function through vesicular transport [24]. Yang et al. demonstrated that plasma lncRNA CoroMarker was mainly stored in EVs, and was a novel biomarker for CAD [25]. However, studies about exosomal lncRNAs on AMI are still sparse. LncRNA MALAT1 is among the most highly expressed lncRNAs that were found to play a crucial role in endothelial cells and atherosclerotic lesion formation [26, 27]. LNC_000226 was a novel lncRNA that was identified in our previous work, and significantly unregulated in the PBMCs of unstable angina patients. In the current study, we examined the expression of MALAT1 and LNC_000226 in circulating exosomes from AMI patients and NCA controls. Our data suggested that both MALAT1 and LNC_000226 were present in plasma exosomes; exosomal LNC_000226 and MALAT1 were potential diagnostic biomarkers for AMI with AUC values of 0.889 and 0.707, respectively.

Although some studies showed that lncRNAs are potential prognostic biomarkers of AMI, the role of exosomal lncRNAs in AMI outcomes is still poorly known. Zheng et al. profiled the exosomal lncRNA expression in AMI patients and found that exosomal lncRNA ENST00000556899.1 and ENST00000575985.1 were potential prognostic biomarkers for AMI [28]. Another study showed that exosomal lncRNA HIF1A‐AS1 was upregulated in atherosclerosis patients and served as a potential biomarker for prognosis prediction [29]. Since LNC_000226 exhibited a better prediction power of AMI in our patient cohort, we investigated its predictive value of AMI outcome. Our data revealed that higher exosomal LNC_000226 levels were linked to an elevated risk of 1‐year MACEs and a lower incidence of MACEs‐free survival in AMI patients. Moreover, exosomal LNC_000226 was an independent factor for MACEs (HR = 1.959). These findings suggest a potential association between LNC_000226 and the prognostic outcomes of AMI.

As circulating exosomes could be derived from various types of cells, e.g. endothelial cells, PBMCs, the sources of exosomal MALAT1 and LNC_000226 are not defined in the present study. Though MALAT1 was found to be upregulated in vascular endothelial cells under hypoxic conditions [30], other studies showed its expression decreased in human atherosclerotic plaques and in the context of inflammatory endothelial cells [26, 27]. LNC_00226 was a novel lncRNA and its expression profile remains to be elucidated across various cell types. Since the expression of MALAT1 and LNC_000226 was significantly increased in PBMCs of patients with unstable angina, it is possible that exosomal MALAT1 and LNC_000226 are derived from PBMCs. However, further work is needed to confirm the sources.

There are some limitations in the present study. It is a single‐center study with a modest sample size, which may affect the generalizability of our findings. The exosomal lncRNAs were detected in blood samples collected from patients during emergency admissions and did not remeasure during the follow‐up, so whether MACE occurrence promotes the secretion of LNC_000226 remains unknown. As for prognosis analysis, 1‐year follow‐up is short, but the follow‐up is continuing, so the association between exosomal LNC_000226 and long‐term outcomes would be clarified in our future work.

## 5 Conclusion

The present study demonstrated that circulating exosomal lncRNA MALAT1 and LNC_000226 are elevated in patients with AMI. Exosomal LNC_000226 was an independent factor for MACEs, and AMI patients with high exosomal LNC_000226 levels suffered a higher risk of 1‐year MACEs. Together, these findings suggested that exosomal MALAT1 and LNC_000226 are potential biomarkers for AMI diagnosis and prognosis.

## Author Contributions

**Xiaodong Gu:** data curation, writing–original draft. **Jingyuan Hou:** methodology, writing–review and editing. **Ruiqiang Weng:** data curation, resources. **Jiawei Rao:** data curation, resources. **Sudong Liu:** project administration, writing–review and editing.

## Ethics Statement

This retrospective observational study was approved by the Ethics Committee of Meizhou People's Hospital (No. MPH‐HEC 2021‐CY‐17).

## Consent

The authors have nothing to report.

## Conflicts of Interest

The authors declare no conflicts of interest.

## Data Availability

The data used is available in the supplementary material and upon reasonable request.
